# IFN-gamma Impairs Release of IL-8 by IL-1beta-stimulated A549 Lung Carcinoma Cells

**DOI:** 10.1186/1471-2407-8-265

**Published:** 2008-09-18

**Authors:** Kim A Boost, Christian D Sadik, Malte Bachmann, Bernhard Zwissler, Josef Pfeilschifter, Heiko Mühl

**Affiliations:** 1 Klinik für Anaesthesiologie, Ludwig-Maximilians-University Munich, Marchioninistr. 15, 81377 Munich, Germany; 2 pharmazentrum frankfurt/ZAFES, Hospital of Johann Wolfgang Goethe-University Frankfurt am Main, Theodor-Stern-Kai 7, 60590 Frankfurt, Germany

## Abstract

**Background:**

Production of interferon (IFN)-γ is key to efficient anti-tumor immunity. The present study was set out to investigate effects of IFNγ on the release of the potent pro-angiogenic mediator IL-8 by human A549 lung carcinoma cells.

**Methods:**

A549 cells were cultured and stimulated with interleukin (IL)-1β alone or in combination with IFNγ. IL-8 production by these cells was analyzed with enzyme linked immuno sorbent assay (ELISA). mRNA-expression was analyzed by real-time PCR and RNase protection assay (RPA), respectively. Expression of inhibitor-κ Bα, cellular IL-8, and cyclooxygenase-2 was analyzed by Western blot analysis.

**Results:**

Here we demonstrate that IFNγ efficiently reduced IL-8 secretion under the influence of IL-1β. Surprisingly, real-time PCR analysis and RPA revealed that the inhibitory effect of IFNγ on IL-8 was not associated with significant changes in mRNA levels. These observations concurred with lack of a modulatory activity of IFNγ on IL-1β-induced NF-κB activation as assessed by cellular IκB levels. Moreover, analysis of intracellular IL-8 suggests that IFNγ modulated IL-8 secretion by action on the posttranslational level. In contrast to IL-8, IL-1β-induced cyclooxygenase-2 expression and release of IL-6 were not affected by IFNγ indicating that modulation of IL-1β action by this cytokine displays specificity.

**Conclusion:**

Data presented herein agree with an angiostatic role of IFNγ as seen in rodent models of solid tumors and suggest that increasing T helper type 1 (Th1)-like functions in lung cancer patients e.g. by local delivery of IFNγ may mediate therapeutic benefit via mechanisms that potentially include modulation of pro-angiogenic IL-8.

## Background

Interleukin (IL)-1β is a cytokine with a key role in the pathophysiology of local and systemic inflammation [1]. Moreover, owing to its pro-inflammatory nature, IL-1β is regarded a tumor-promoting cytokine. In fact, enhanced tumor metastasis and angiogenesis has been observed under the influence of IL-1β [2,3]. Accordingly, IL-1β is able to facilitate tumor progression in murine models of lung cancer. Upregulation of metastasis and tumor angiogenesis by IL-1β as observed in those studies was associated with increased activity of matrix metalloproteinases and expression of the pro-angiogenic molecule hepatocyte growth factor. Furthermore, blockage of the chemokine receptor CXCR2 inhibited tumor growth *in vivo *indicating that a functional murine IL-8 homologue contributes to IL-1β-mediated progression of disease [4,5]. Notably, the chemokine IL-8 (CXCL-8) is an efficient mediator of angiogenesis [6,7] and thus located at the crucial interface of inflammation and tumor biology. Neutralization of IL-8 reduced tumorigenesis of human non-small cell lung cancer (NSCLC) in the SCID mouse model [8]. A key role for IL-1β-inducible IL-8 in the progression of lung cancer is strongly suggested by various clinical studies demonstrating that IL-8 detected in patient biopsy specimens positively correlates with tumor angiogenesis and metastasis. Moreover, IL-8 is associated with shortened survival, particularly in NSCLC [9-12]. Cell culture data suggest that lung carcinoma cells are a highly relevant source of IL-8 in the tumor microenvironment [9,13]. Interestingly, a recent study also demonstrates that IL-8 mediates proliferation of the human NSCLC cell lines A549 and NCI-H292, respectively [14]. Those observations further underscore that IL-8 can be regarded a pivotal factor in the progression of lung cancer.

Bulk of data from preclinical research indicates that interferon (IFN)-γ mediates important tumor-suppressive functions. Those include supression of proliferation and angiogenesis, induction of apoptosis, and activation of leukocytes with anti-cancer activity such as NK cells, NKT cells and T cells [15]. Interestingly, inhaled IFNγ showed therapeutic efficacy in a murine model of lung cancer [16]. Moreover, application of IFNγ by aerosol is able to activate alveolar macrophages in human beings [17] and shows therapeutic potential in tuberculosis patients [18,19]. In order to further characterize IL-8 as an immunopharmacological target, we set out to investigate in the present study effects of the tumorsuppressive Th1-like cytokine IFN-γ on the production of IL-8 by NSCLC A549 cells under the influence of IL-1β.

## Methods

###  Cell Culture

Human A549 lung carcinoma/epithelial cells were obtained from the German Collection of Microorganisms and Cell Cultures (Braunschweig, Germany). Cells were maintained in RPMI 1640 supplemented with 10 mM HEPES, 100 U/ml penicillin, 100 μg/ml streptomycin, and 10% heat-inactivated FCS (GIBCO-BRL, Eggenstein, Germany). For the experiments, confluent cells on polystyrene plates (Greiner, Frickenhausen, Germany) were washed with PBS and incubated in the aforementioned medium. Human IFNγ was obtained from TEBU/Peprotech (Frankfurt, Germany) and IL-1β from Invitrogen/Biosource (Karlsruhe, Germany).

###  Detection of IL-8 and IL-6 by enzyme-linked immunosorbent assay (ELISA)

Levels of IL-8 and IL-6 in cell-free culture supernatants were determined by ELISA according to the manufacturer's instructions (BD Bioscience/Pharmingen, Heidelberg, Germany). Two splice variants of IL-8 with different C-terminal regions can be discriminated (database Swiss-Prot/TrEMBL, ). According to information provided by the manufacturer both splice variants of IL-8 are being detected by the ELISA assay used in the current study. In addition, various N-terminal IL-8 variants that are being generated by proteolytic cleavage are recognized by the assay.

###  Analysis of IL-8 and glyceraldehyde-3-phosphate-dehydrogenase (GAPDH) mRNA expression by real-time polymerase chain reaction (PCR) analysis, standard PCR analysis and RNase protection assay (RPA)

After RNA isolation (peqGold TriFast, Peqlab, Erlangen, Germany), 1 μg of total RNA was transcribed using random hexameric primers and Moloney virus reverse transcriptase (RT) (Applied Biosystems, Darmstadt, Germany). During real-time PCR analysis changes in fluorescence are caused by the Taq-polymerase degrading the probe that contains a fluorescent dye (FAM used for IL-8, VIC used for GAPDH) and a quencher (TAMRA). For IL-8 (Hs00174103_m1) and GAPDH (4310884E) pre-developed assay reagents were obtained from Applied Biosystems. Assay-mix was purchased from Invitrogen (Karlsruhe, Germany). Real-time PCR was performed using the AbiPrism 7700 Sequence Detector (Applied Biosystems) as follows: One initial step at 50°C for 2 minutes and 95°C for 2 minutes was followed by 40 cycles at 95°C for 15 seconds and 60°C for 1 minute. Detection of the dequenched probe, calculation of threshold cycles (Ct values), and further analysis of these data were performed by the Sequence Detector software. Relative changes in IL-8 mRNA expression compared to unstimulated control and normalized to GAPDH were quantified by the 2^-ddCt ^method.

For detection of both IL-8 splice forms 1 μg of RNA was used for standard RT-PCR (Applied Biosystems, Darmstadt, Germany) with GoTaq DNA polymerase (Promega, Mannheim, Germany). The following sequences were performed for PCR: 94°C for 10 min (1 cycle); 95°C for 30 s, 59°C for 30 s, and 72°C for 1 min (with variable numbers of cycles); extension phase at 72°C for 7 min. Numbers of cycles: GAPDH, 24; IL-8, as indicated. Sequences of the primers and length of resulting amplicons: IL-8 (F) 5'-atgacttccaagctggcc gtggct-3'; IL-8 (R1): 5'-ttatgaattctcagccctcttcaaaaa-3' (detects the dominat form of IL-8), 299 bp; IL-8 (R2): 5'-ccctgttttcagggacctctgc-3' (detects the minor/rare form of IL-8, this form is denoted as IL-8 variant in the figures in the results, 294 bp; GAPDH (F): 5'-accacagtccatgccatcac-3', GAPDH (R): 5'-tccaccaccctgttgctgta-3', 452 bp.

10 μg of total RNA were used for RPA analysis of IL-8 mRNA expression. DNA probes were cloned into the transcription vector pBluescript II KS (+) (Stratagene, Heidelberg, Germany). After linearization, an antisense transcript was synthesized *in vitro *by T7 RNA polymerase and [α-^32^P]UTP (800 Ci/mmol). RNA samples were hybridized at 42°C overnight with 50,000 c.p.m. of the labeled antisense transcript. Hybrids were digested by RNase A and RNase T1 (Roche) for 1 hour at 30°C. Under these conditions every single mismatch was recognized by the RNases. Protected fragments were separated on 5% (w/v) polyacrylamide/8 M urea gels and analyzed using a PhosphoImager device (Fuji, Straubenhardt, Germany). Individual expression of IL-8 was evaluated on the basis of the GAPDH housekeeping gene expression and is shown as n-fold induction. The cloned cDNA probes for detection of IL-8 and GAPDH correspond to nucleotides (nt) 16–270 and nt 961–1071 of the published sequences (hIL-8, NM000584.2; hGAPDH, AC M33197).

### Detection of inhibitor of κBα(IκBα), cyclooxygenase-2 (COX-2), IL-8, and β-actin by Western blot analysis

A549 cells were harvested using lysis buffer (150 mM NaCl, 1 mM CaCl_2_, 25 mM TrisCl, pH 7.4, 1% Triton-X-100, supplemented with protease inhibitor cocktail (Roche Diagnostics, Mannheim, Germany) and DTT, Na_3_VO_4_, PMSF (each 1 mM) and NaF (20 mM)). 50 μg of total protein/lane were used. Antibodies and SDS-PAGE conditions: IκBα, 12% SDS-PAGE, polyclonal antibody (Santa Cruz Biotechnology); COX-2, 10% SDS-PAGE, monoclonal antibody (Santa Cruz Biotechnology); IL-8, 18% SDS-PAGE, monoclonal antibody (R&D Systems, Wiesbaden, Germany); for detection of β-actin (monoclonal antibody, Sigma) blots were stripped and reprobed. For detection of intracellular IL-8, cells were incubated with Brefeldin A (BfA) at 10 μg/ml (Sigma, Hamburg, Germany) in order to suppress the cellular secretory machinery. In those experiments all conditions were adjusted to a final concentration of 0.05% DMSO in order to control for the BfA vehicle.

###  Statistics

Data are shown as mean ± SD and are presented as pg/ml, ng/ml, or as fold-induction compared to unstimulated control. Data were analyzed by unpaired Student's t test on raw data using Sigma PLOT/STAT (Jandel Scientific).

## Results

###  IFNγ impairs release of IL-8 from IL-1β-stimulated A549 cells

**Figure 1:**
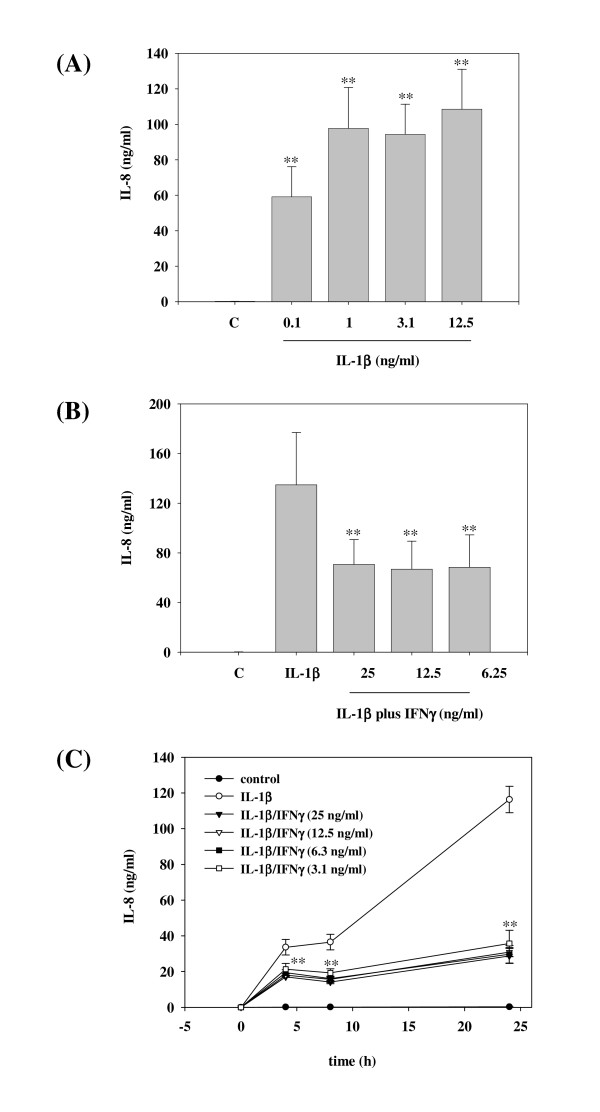
**IFNγ modulates release of IL-8 by IL-1β-stimulated A549 cells.(A) **A549 cells were incubated as unstimulated control, or stimulated with the indicated concentrations of IL-1β. After 24 h, cell-free supernatants were assayed for IL-8 protein content by ELISA analysis. Data are expressed as mean IL-8 concentrations ± SD (n = 4). **p < 0.01 compared with untreated control. **(B) **A549 cells were incubated as unstimulated control, stimulated with IL-1β (25 ng/ml), or with IL-1β (25 ng/ml) in combination with the indicated concentrations of IFNγ. After 24 h, cell-free supernatants were assayed for IL-8 content by ELISA analysis. Data are expressed as mean IL-8 concentrations ± SD (n = 8). **p < 0.01 compared with IL-1β alone. **(C) **A549 cells were incubated as unstimulated control or were stimulated with IL-1β (25 ng/ml). Where indicated, A549 cells were preincubated for 16 h with IFNγ at different concentrations (ranging from 3.1 ng/ml up to 25 ng/ml). IL-1β was added directly thereafter to the cultures without further washing. After the indicated time periods with or without IL-1β stimulation, cell-free supernatants were assayed for IL-8 content by ELISA analysis. Data are expressed as mean IL-8 concentrations ± SD (n = 7). **p < 0.01 (for IFNγ at 3.1 ng/ml) compared with IL-1β alone.

In accord with previous reports [20] we observed strong induction of IL-8 secretion by A549 cells under the influence of IL-1β. A saturation range was reached at an IL-1β concentration of 1 ng/ml (Figure 1A). IL-8 release could not be further enhanced, even by increasing the dose of IL-1β to 25 ng/ml or 50 ng/ml, respectively (data not shown). In the present study we sought to investigate a potential regulatory role of IFNγ concerning IL-1β-induced IL-8 in A549 cells. All subsequent experiments were performed by using high saturating IL-1β concentrations (≥ 1 ng/ml) in order to ensure complete pro-inflammatory activation of those cells and thus to set a high hurdle for modulation of IL-8 production by anti-inflammatory intervention. As shown in Figure 1B, coincubation with IFNγ significantly impaired release of IL-8 from IL-1β-stimulated A549 cells during a 24 h incubation period. IFNγ as a single stimulus was unable to mediate IL-8 release by A549 cells (data not shown). Figure 1C demonstrates that preincubation of A549 cells with IFNγ for 16 h even more enhanced this inhibitory action of IFNγ on IL-1β-induced IL-8 release. Notably, the modulatory function of IFNγ was already detectable after a 4 h incubation period with IL-1β (Figure 1C).

###  The modulatory function of IFNγ on IL-8 secretion is not associated with changes in cellular IL-8 mRNA or protein expression

**Figure 2:**
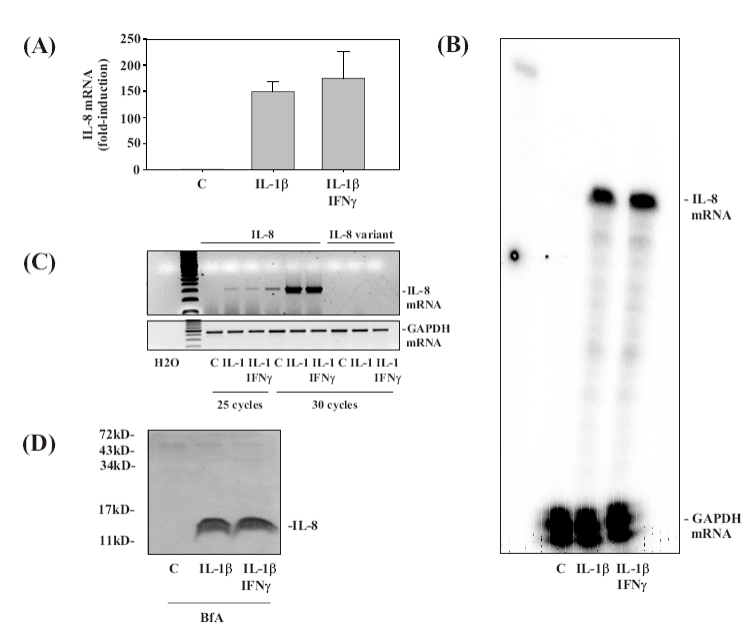
**IFNγ is unable to modulate IL-8 mRNA and protein expression under the influence of IL-1β.** A549 cells were incubated as unstimulated control, or stimulated with IL-1β (1 ng/ml). Where indicated, A549 cells were preincubated for 16 h with IFNγ at 20 ng/ml. IL-1β was added directly thereafter to the cultures without further washing. After 10 h, IL-1β-induced IL-8 mRNA accumulation was evaluated by realtime PCR analysis **(A) **and RPA **(B)**, respectively. IL-8 mRNA expression was normalized to that of GAPDH. **(A) **Data are expressed as fold-induction compared to unstimulated control ± S.D. (n = 3). IL-8 protein levels in cell-free culture supernatants of those same cultures were determined by ELISA analysis (see results section). **(B) **One representative of three independently performed RPA analyses is shown. **(C) **RNA populations were analyzed by standard PCR using primers pairs that specifically detect the two IL-8 splice variants. The minor/rare splice form of IL-8 is denoted as 'IL-8 variant'. **(D) **A549 cells were incubated as unstimulated control, or stimulated with IL-1β (1 ng/ml). Where indicated, A549 cells were preincubated for 16 h with IFNγ at 20 ng/ml. Furthermore, 1 h before stimulation with IL-1β, BfA (10 μg/ml) was added to all cultures in order to block the cellular secretory machinery. After 8 h of incubation with IL-1β, cells were harvested and cellular IL-8 protein expression was assessed by Western blot analysis. One representative of three independently performed experiments is shown.

Since reduction of IL-8 mRNA expression is an appealing potential mechanism responsible for the inhibitory IFNγ action observed herein, real-time PCR analysis for IL-8 gene expression was performed (Figure 2A). Notably, despite thorough investigation we were unable to detect significant changes of IL-8 mRNA levels under the influence of IFNγ. In this same set of experiments IL-1β-induced IL-8 protein release (10 h stimulation period) was inhibited by IFNγ by 52.3% ± 5.0% compared to IL-1β alone (set as 100%). A lack of an IFNγ effect on the level of IL-8 mRNA expression was also obtained using RNase protection assay as an alternative method for quantification of mRNA populations (Figure 2B). Although both splice variants of IL-8 (Swiss-Prot/TrEMBL, ) would be recognized by the ELISA used herein, we sought to investigate whether IL-8 splicing is affected by coincubation of A549 cells with IFNγ. For that purpose standard PCR analysis was performed using primers pairs that specifically discriminate between the different C-termini of both IL-8 forms. Figure 2C demonstrates that we were unable to detect the minor/rare splice form of IL-8 (denoted as IL-8 variant) in A549 cells, irrespective of the presence or absence of IFNγ.

In order to further characterize the mechanism of IFNγ action on IL-1β-induced IL-8 production, secretion of the cytokine was blocked by coincubation with BfA. Western blot analysis under those condition clearly demonstrated that translation of IL-8 mRNA into protein is not affected by IFNγ (Figure 2D).

###  IFNγ is not a general inhibitor of IL-1β action on A549 cells

**Figure 3:**
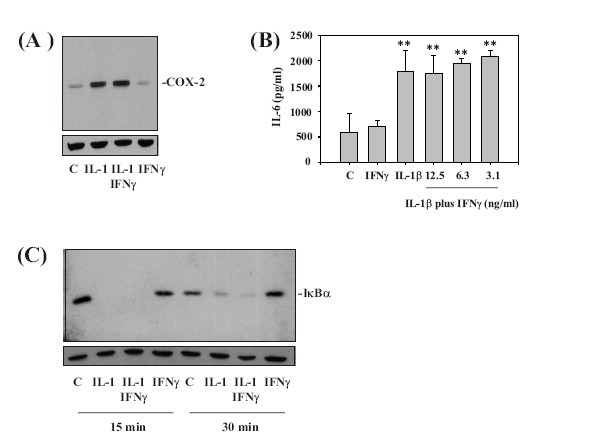
**Analysis of IL-6 release, COX-2 expression, and IκB degradation reveals that IFNγ can not be regarded as a general inhibitor of IL-1β action on A549 cells.(A) **A549 cells were incubated as unstimulated control, or stimulated with IL-1β (12.5 ng/ml). Where indicated, A549 cells were preincubated for 16 h with IFNγ at 20 ng/ml. IL-1β was added directly thereafter to the cultures without further washing. After 24 h, cell-lysates were assayed for COX-2 expression by Western blot analysis. One representative of three independently performed experiments evaluating COX-2 expression is shown. **(B) **A549 cells were incubated as unstimulated control, or stimulated with IL-1β (12.5 ng/ml). Where indicated, A549 cells were preincubated for 16 h with either IFNγ at 12.5 ng/ml (without later addition of IL-1β) or with the indicated concentrations of IFNγ in combination with IL-1β (12.5 ng/ml). IL-1β was added directly thereafter to the cultures without further washing. After 24 h, cell-free supernatants were assayed for IL-6 secretion by ELISA. Data are expressed as mean IL-6 concentrations ± SD (n = 3). **p < 0.01 compared with untreated control. **(C) **A549 cells were incubated as unstimulated control or stimulated with IL-1β (12.5 ng/ml). Where indicated, A549 cells were preincubated for 16 h with IFNγ at 20 ng/ml. IL-1β was added directly thereafter to the cultures without further washing. After 15 min or 30 min, cells were harvested and homogenates were evaluated for IκBα-protein by Western blot analysis. One representative for three independently performed experiments is shown.

In order to investigate whether IFNγ should be regarded as a general inhibitor of IL-1β action on A549 cells, COX-2 (Figure 3A) and IL-6 (Figure 3B) were investigated as additional prototypic IL-1β-inducible proteins. Interestingly, Western blot and ELISA analysis revealed that expression/release of neither parameter was significantly affected by IFNγ in the context of IL-1β-activated A549 cells. Those data concur with the further observation that IFNγ was likewise unable to modulate degradation of IκBα in response to IL-1β, indicating that IFNγ left activation of nuclear factor-κB (NF-κB) under those conditions unaffected (Figure 3C). In addition to that, experiments using conditioned media from IFNγ-stimulated A549 cells also excluded the possibility that IFNγ mediates production of an 'IL-8 binding protein' that might have impaired IL-8 detection by ELISA (data not shown).

## Discussion

Impaired production of Th1-like cytokines and/or enhanced expression of Th2-like cytokines and IL-10 has been associated with tumor progression in a variety of malignancies, including lung cancer [21-23]. Modulation of angiogenesis appears to be a prime mechanism by which anti-cancer immunity restrains growth of solid tumors [24]. Specifically, production of the IFNγ-inducible angiostatic non-ELR^+^chemokines CXCL-9 (MIG), CXCL-10 (IP-10), and CXCL-11 (I-TAC) is of key relevance in this context [6]. Accordingly, production of CXCL-9 and CXCL-10 has been associated with tumorsuppression in animal models of NSCLC [25,26]. In the present study we demonstrate for the first time that IFNγ has the capability to significantly inhibit secretion of the pro-angiogenic chemokine IL-8 by A549 NSCLC cells. It is important to bear in mind that IL-8 not only is a mediator of angiogenesis but is obviously a crucial chemoattractant for neutrophils [27]. In this context it is of interest that lung carcinogenesis driven by inflammation has been associated with influx of neutrophils into the airway compartment [28]. The current observation concurs with distinct anti-inflammatory properties of immunoregulatory IFNγ [29] that include modulation of IL-8 secretion as previously noted for other human cell types including monocytes/macrophages [30], synoviocytes [31], and melanoma cells [32]. Effects of IFNγ on IL-8 were not of unspecific nature but displayed discrete specificity since IL-1β-induced expression/secretion of COX-2 and IL-6 were left unaffected. Analysis of cellular IL-8 mRNA and protein expression furthermore revealed that effects of IFNγ on IL-8 release by A549 cells do not affect IL-8 production but are obviously mediated by an unforeseen mechanism that targets the process of secretion and warrants further investigation.

Generally speaking, clinical trials investigating the therapeutic potential of IFNγ in cancer had a disappointing outcome [33]. However, one phase II clinical study suggests that a subgroup of NSCLC patients may benefit from therapeutic intravenous application of IFNγ in combination with chemotherapy. This trend observed did not reach statistical significance, possibly due to the fact that numbers of patients included in that trial were limited (26 for chemotherapy alone *versus *27 for chemotherapy plus IFNγ). Moreover, an increased incidence of hematological toxicity was evident in the study arm undergoing intraveneous IFNγ treatment [34]. In the context of lung pathology application by aerosol might be of considerable advantage with regard to the therapeutic potential of IFNγ. Delivery of IFNγ by inhalation represents a route of administration that achieves high local concentrations at the lung along with reduced systemic toxicity in human beings [35]. In fact, this strategy proved promising in a murine model of lung cancer [16]. Moreover, inhalation of IFNγ has already been suggested for the treatment of tuberculosis and appears to show clinical efficacy [18,19]. In light of the current data and regarding previously published information delineating the tumorsuppressive potential of IFNγ it is tempting to speculate that inhaled IFNγ may pharmacologically differ from systemically applied and may indeed act as an immunostimulatory/-regulatory adjuvant with the potential to provide therapeutic benefit.

## Conclusion

Taken together, data presented herein suggest that the Th1 signature cytokine IFNγ may not only be able to modulate angiogenesis in the NSCLC microenvironment by increasing the production of anti-angiogenic non-ELR^+ ^chemokines. Concurrently, IFNγ clearly has the potential to suppress the production of pro-angiogenic IL-8 by A549 NSCLC carcinoma cells. This observation might be of translational relevance as clinical studies identified IL-8 as being a factor that is strongly associated with reduced survival of NSCLC patients [9,10].

## Competing interests

The authors declare that they have no competing interests.

## Authors' contributions

KAB and CDS performed the cell culture, ELISA analysis, RPAs, and Western blot analyses. CDS and MB performed PCR-analyses. HM, JP, BZ and KAB designed the study and drafted out the manuscript. All authors have read and approved the manuscript.

## Pre-publication history

The pre-publication history for this paper can be accessed here:


